# Gut macrobiotic and its metabolic pathways modulate cardiovascular disease

**DOI:** 10.3389/fmicb.2023.1272479

**Published:** 2023-09-26

**Authors:** Junwen Zhu, Jin Lyu, Ruochi Zhao, Gang Liu, Shuangshuang Wang

**Affiliations:** ^1^Department of Cardiology, The Affiliated Wenling Hospital of Wenzhou Medical University (The First People’s Hospital of Wenling), Zhejiang, China; ^2^College of Bioscience and Biotechnology, Hunan Agricultural University, Changsha, Hunan, China; ^3^Department of Pathology, The First People’s Hospital of Foshan, Foshan, Guangdong, China; ^4^Key Laboratory of Precision Medicine for Atherosclerotic Diseases of Zhejiang Province, Affiliated First Hospital of Ningbo University, Ningbo, China

**Keywords:** cardiovascular disease, gut microbiota, metabolites, host immune, hypertensive

## Abstract

Thousands of microorganisms reside in the human gut, and extensive research has demonstrated the crucial role of the gut microbiota in overall health and maintaining homeostasis. The disruption of microbial populations, known as dysbiosis, can impair the host’s metabolism and contribute to the development of various diseases, including cardiovascular disease (CVD). Furthermore, a growing body of evidence indicates that metabolites produced by the gut microbiota play a significant role in the pathogenesis of cardiovascular disease. These bioactive metabolites, such as short-chain fatty acids (SCFAs), trimethylamine (TMA), trimethylamine N-oxide (TMAO), bile acids (BAs), and lipopolysaccharides (LPS), are implicated in conditions such as hypertension and atherosclerosis. These metabolites impact cardiovascular function through various pathways, such as altering the composition of the gut microbiota and activating specific signaling pathways. Targeting the gut microbiota and their metabolic pathways represents a promising approach for the prevention and treatment of cardiovascular diseases. Intervention strategies, such as probiotic drug delivery and fecal transplantation, can selectively modify the composition of the gut microbiota and enhance its beneficial metabolic functions, ultimately leading to improved cardiovascular outcomes. These interventions hold the potential to reshape the gut microbial community and restore its balance, thereby promoting cardiovascular health. Harnessing the potential of these microbial metabolites through targeted interventions offers a novel avenue for tackling cardiovascular health issues. This manuscript provides an in-depth review of the recent advances in gut microbiota research and its impact on cardiovascular health and offers a promising avenue for tackling cardiovascular health issues through gut microbiome-targeted therapies.

## 1. Introduction

Cardiovascular disease has emerged as a leading cause of morbidity and mortality worldwide (Writing Group Members, 2016). The World Health Organization (WHO) recognizes CVD, encompassing conditions like hypertension, atherosclerosis, heart failure, coronary heart disease, and cerebrovascular disease, as a major public health concern. In 2017 alone, CVDs claimed the lives of approximately 17.8 million individuals, accounting for 31% of the global mortality rate (WHO CVD Risk Chart Working Group, 2019). This staggering figure surpasses that of any other cause of death, reflecting the urgent need to address and mitigate the impact of CVDs.

The gut microbiome performs a crucial function in maintaining the health and stability of the human body and exhibits a symbiotic relationship with its host. The gut microbiota consists of trillions of microorganisms that have complex interactions with the host (Krautkramer et al., 2017), besides major consists of the phylum Firmicutes, Bacteroidetes, and Actinobacteria (The Human Microbiome Project Consortium, 2012). Current research suggests that variations in the gut microbiome might be strongly associated with the evolution of cardiovascular disease (Karlsson et al., 2012). Gut microbiota of atherosclerosis patients were found to be elevated in abundance in *Collinsella genus*, *Enterobacteriaceae*, *Streptococcaceae*, and *Klebsiella* spp. compared to healthy individuals, whereas SCFA-producing bacteria *Eubacterium*, *Roseburia*, and *Ruminococcaceae* spp. decreased in abundance (Jie et al., 2017). Researches have found that shifts in the composition of the gut microbiota, as well as its metabolites, could exacerbate the progression of cardiovascular disease (Vinje et al., 2014). Recent studies relating to patients with coronary artery disease have highlighted marked increases in populations of some bacteria in the gut, while the number of beneficial bacteria like *Bacteroidetes* and *Lactobacillus* are reduced (Verhaar et al., 2020). These alterations in gut flora may promote the progression of atherosclerosis by causing an increased inflammatory response.

In addition, gut flora-derived metabolites have been shown to be contributing factors to disease development. Some metabolites with potential roles in atherosclerosis and hypertension include short-chain fatty acids, trimethylamine, trimethylamine N-oxide, bile acids, and lipopolysaccharides (Seldin et al., 2016). These metabolites as an essential role in the progression of cardiovascular disease by influencing aspects of the host’s immune response, inflammatory response, and vascular functions. For instance, in a study involving wild-type NMRI mice infused with propionic acid-treated Ang II, propionate significantly attenuated cardiac hypertrophy, fibrosis, vascular dysfunction, hypertension, and systemic inflammation (Bartolomaeus et al., 2019). To elucidate the specific relationship between gut microbes and cardiovascular disease, the link between CVD pathogenesis and gut flora also needs to be investigated to provide further evidence that controlling gut disorders may be able to serve as a CVD preventive and therapeutic strategy. In this review, we describe the relevance of the gut microbiota and its derived metabolites to cardiovascular disease and their therapeutic potential for the treatment of CVD.

## 2. Metabolic pathways and products of the intestinal macrobiotic

### 2.1. Short chain fatty acids

**Figure 1:**
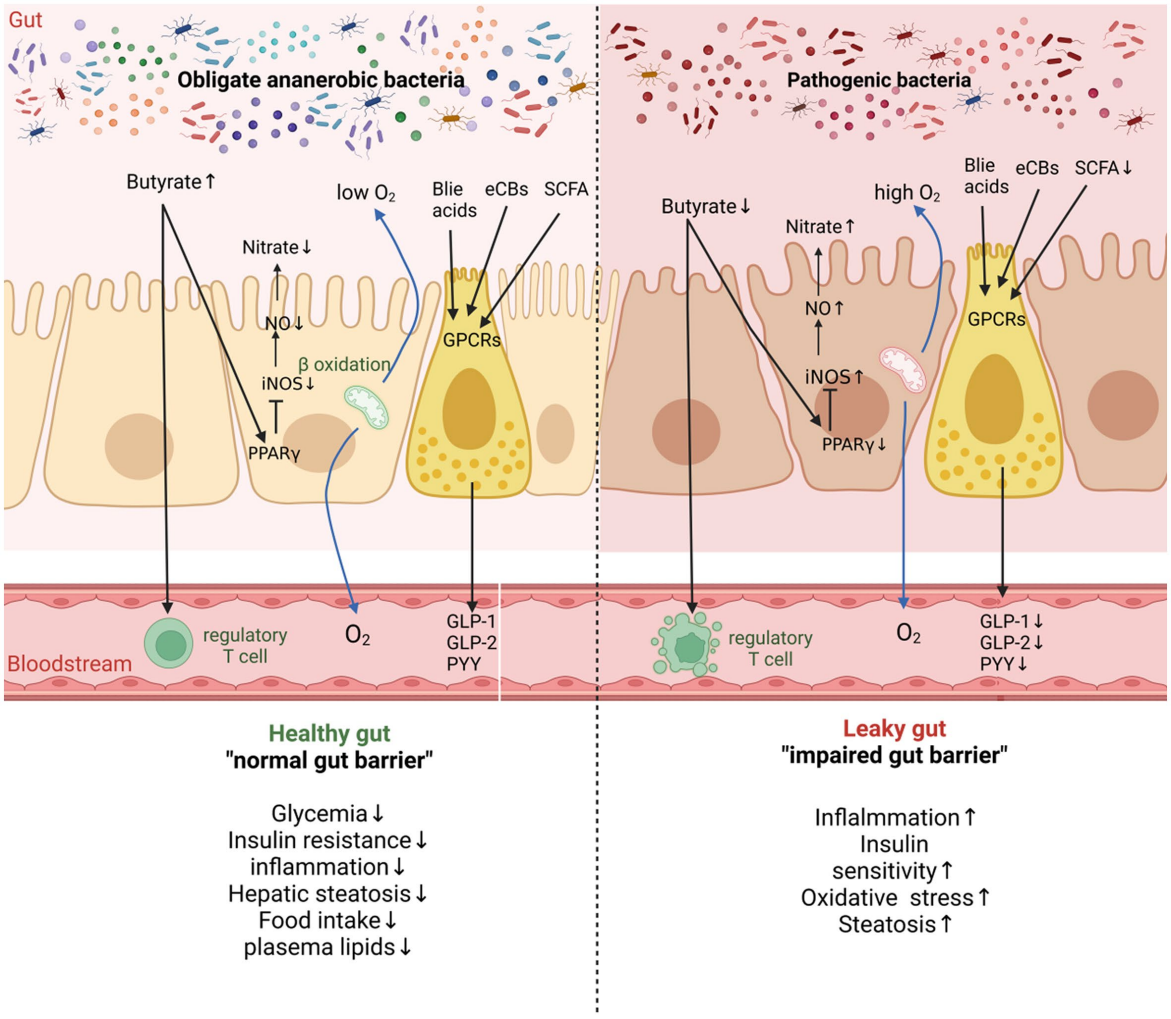
The molecular mechanisms linking the gut macrobiotic and host 262 health in healthy and pathological conditions. In healthy conditions, the intestinal 263 lumen is maintained in a constant anaerobic state by oxidation in the mitochondria. In 264 pathological conditions, the intestinal macrobiota is regulated and decreased levels of 265 butyrate lead to inflammation in T cells, resulting in impaired intestinal barrier 266 function, which in turn affects the metabolic processes of the host. Butyrate promotes 267 iNOS production and enhances the nitrate content by binding to peroxisome 268 value-added activated receptor γ (PPARγ). SCFAs, BAs and others act on GPCRs 269 receptors and regulate lipid metabolism.

Short-chain fatty acids (SCFAs) are a major group of metabolites produced by intestinal microorganisms. They are primarily generated in the colon from the fermentation of probiotic dietary fibers (Cummings et al., 1987), which are beneficial to bacterial activities. SCFAs can be classified into acetic acid, propionate, and butyrate, with acetic acid being the most abundant SCFA produced by intestinal microorganisms (Cummings et al., 1979), which are mostly created by bacteria including *Bifidobacterium bifidum* and *Lactobacillus lactis* (Parada Venegas et al., 2019). Acetic acid helps to regulate the acid–base balance of the gut and maintains the stability of the internal environment (Rosenstein et al., 1981). Propionate, another SCFA, provides health benefits such as lowering cholesterol, reducing fat storage, exhibiting anti-cancer properties and possessing anti-inflammatory effects as a result of *colonic bacteria* fermentation (Hosseini et al., 2011). Butyrate is mainly produced by anaerobic bacteria like *Eubacterium rectal* and *Faecalibacterium* and serves as an important energy source in the colon (Chen et al., 2020). *Eubacterium rectal* and *Faecalibacterium* make up about 10% of the healthy gut. It also has a significant effect in fighting inflammation (Hafidi et al., 2019). SCFAs as a pivotal key in maintaining the ecological balance of gut microbes, fine-tuning the host immune system, and managing metabolic diseases (Figure 1; Yang et al., 2018). Butyrate activates a series of G protein-coupled receptors (GPCRs) that affect key metabolic processes, and GPCR41 and GPCR43 are expressed in immune cells (Kimura et al., 2013). Furthermore, they are instrumental in maintaining intestinal health and preventing related diseases (Koh et al., 2016).

### 2.2. Trimethylamine and trimethylamine N-oxide

Trimethylamine N-oxide (TMAO) is a metabolite derived from intestinal flora that has been extensively studied in humans and mice. It is primarily produced through the metabolism of dietary choline, phosphatidylcholine, and carnitine by intestinal flora, resulting in the generation of trimethylamine (TMA). TMA is then oxidized to TMAO by the enzyme Riboflavin-containing monooxygenase 3 (FMO3) in the liver (Naghipour et al., 2021).

Elevated levels of TMAO have been identified as a significant risk factor for the development of cardiovascular diseases such as atherosclerosis and thrombosis (Senthong et al., 2016; Roncal et al., 2019). Accelerated atherosclerotic plaque formation in mice has been demonstrated in a mouse model study of dietary supplementation with TMAO or its precursors (Zhao et al., 2018). By measuring TMAO levels in plasma from healthy individuals and patients with cardiovascular disease, it was found to be 4.04 μM in patients with cardiovascular disease, whereas plasma levels of TMAO were as low as 3.21 μM in healthy individuals (Gatarek and Kaluzna-Czaplinska, 2021). Furthermore, a high TMAO level is considered an indicator of an increased disease risk in the future (Canyelles et al., 2018). This association has been supported by numerous experimental and clinical data, which have also shown that TMAO can accelerate the progression of atherosclerosis in animal models (Wang et al., 2011; Tang et al., 2013). For example, C57BL/6 J, Apo^−/−^ mice were fed regular chow versus the same chow supplemented with L-carnitine, respectively, from the time of weaning. In contrast, Apoe^−/−^ mice had an approximately doubled atherosclerotic burden and elevated plasma levels of TMA and TMAO (Koeth et al., 2013). And another study showed by 16S rRNA sequencing that Apoe^−/−^ mice fed a high choline diet had a 44.2% higher burden of atherosclerosis than mice with *M. smithii* (Ramezani et al., 2018). Several reviews in recent years have also emphasized the clinical relevance of the TMAO pathway in cardiovascular disease, highlighting its potential as a treatment target (Brown and Hazen, 2015). Several studies have demonstrated a clear association between intestinal flora and cardiovascular disease, providing valuable insights for further exploration of the potential of the TMAO pathway in the prevention and treatment of CVD (Tang and Hazen, 2014).

### 2.3. Bile acids

Bile acids are an important class of biologically active molecules that play a vital role in the human body. They include primary bile acids, secondary bile acids, cholic acid (CA), and chenodeoxycholic acid (CDCA; Ticho et al., 2019). Primary bile acids are synthesized in the liver through monochrome P450-mediated cholesterol oxidation (de Aguiar Vallim et al., 2013), Bile acids connect the liver, the intestinal microbiota, and the gut, with the microbiota converting primary bile acids into secondary bile acids (Hosseinkhani et al., 2021). These secondary bile acids promote the uptake of dietary fatty acids and activate cell surface signaling and upregulate nuclear hormone receptor levels. Recent studies have shown that the mammalian gut microbiota is involved in the regulation of bile acid synthesis and metabolism (Jones et al., 2008), and that BAs can be further modified by specific enzymes in the gut microbiota, such as bile salt hydrolase (BSH) and bile acid dehydratase. BSH cleaves the amide bonds of glycine and taurine that are partially bound to the nucleus of bile salts and steroid nuclei and releases bile acids (Long et al., 2017).

**Figure 2:**
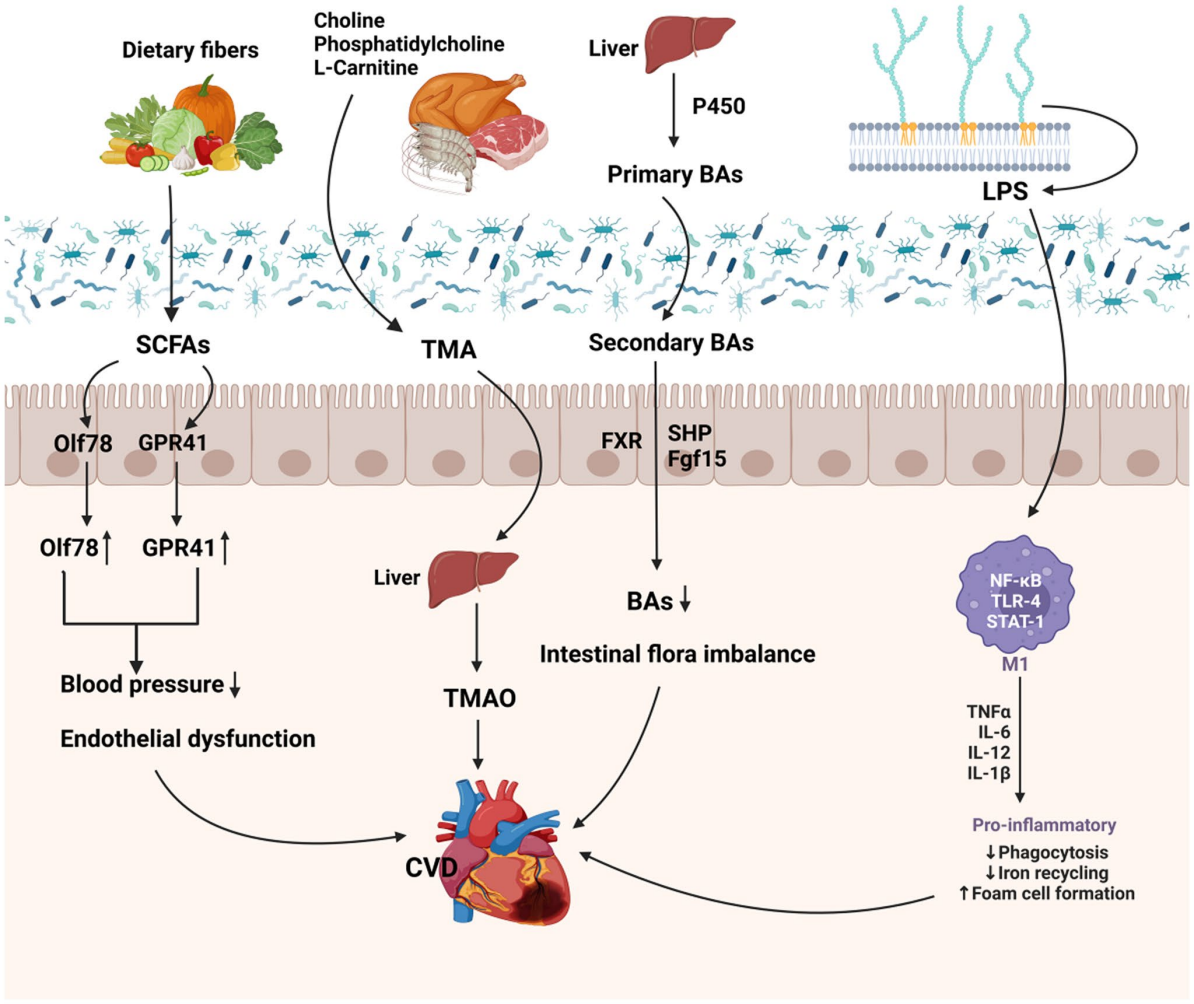
Potential pathogenesis of dietary metabolites of intestinal flora 185 in CVD. SCFA, produced mainly from dietary fiber, causes a dramatic drop in blood 186 pressure by binding to two receptors, Olf78 and GPR41. TMA is oxidized in the liver 187 to become TMAO, TMAO has been shown to promote the development of 188 atherosclerosis and increase the risk of cardiovascular disease. Primary bile acids are 189 catalyzed by monochrome P450 in the liver to oxidize cholesterol to secondary bile 190 acids, resulting in intestinal disorders. LPS promotes the release of inflammatory 191 factors by activating the NF-κB, toll-like receptors (TLRs) and STAT-1 signaling 192 pathways, thereby inducing inflammation.

The interaction between bile acids and the gut microbiota has significant implications for the maintenance of intestinal health, regulation of cholesterol metabolism, and the function of the gut barrier. Recent studies have shown that the intestinal absorption of cholesterol can be inhibited and plasma cholesterol levels can be reduced by regulating bile acid metabolism (Westin et al., 2005; Xu and Yang, 2020). Research has shown that BAs in combination with its activating transcription factor, farnesoid X receptor (FXR), inhibit cholesterol transport and increase cholesterol excretion by macrophages in order to reduce cholesterol concentration in plasma, and can be used as a therapeutic target for dyslipidemia (Figure 2; Kawamata et al., 2003; Westin et al., 2005). Thus, understanding the complex links between the gut microbiota, bile acids, and cardiovascular health is crucial (Chen et al., 2016). These findings also open up new avenues for further research on the association between the gut microbiota and cardiovascular diseases (Xu and Yang, 2020).

### 2.4. Lipopolysaccharide

Lipopolysaccharide is a specialized ingredient found in the outer membrane of Gram-negative bacteria (Maldonado et al., 2016). *In vivo*, LPS is able to activate various cell types, consisting of monocyte macrophages, endothelial cells, and epithelial cells, through cell signaling systems (Gabarin et al., 2021). Activated cells synthesize and release a variety of cytokines and inflammatory mediators, as well as activate signaling pathways such as NF-κB (Hailman et al., 1994), MAPK (Chang et al., 2019), toll-like receptors (TLRs) and STAT-1, which triggers the synthesis and release of cytokines and inflammatory mediators like interleukin-1 (IL-1), interleukin-6 (IL-6), tumor-necrosis factor-α (TNF-α; Wright et al., 1990; Mehta et al., 2012), and nitric oxide (NO). This causes an inflammatory response. LPS can affect intestinal permeability, disrupting the intestinal barrier and allowing other bacterial components and LPS to enter the circulation and trigger a systemic inflammatory response (Cani and Delzenne, 2010). At the same time, LPS also can directly interact with host cells via TLRs to regulate the inflammatory responses (Mehta et al., 2010; Larsson et al., 2012). LPS-induced inflammatory responses have been found to be tied to several metabolic disorders, including hyperinflation (high blood glucose; Gomes et al., 2017), obesity and fatty liver (Lepper et al., 2007; Amar et al., 2008). Several studies have found that certain fatty acids can bind to LPS, thereby attenuating the inflammatory response it induces and ameliorating the associated metabolic abnormalities (Hutchinson et al., 2020).

## 3. Gut macrobiotic and cardiovascular disease

### 3.1. Gut macrobiotic and hypertension

Hypertension constitutes among the most prevalent CVDs (Yang et al., 2023), which is caused by a wide range of environmental and genetic factors (Kato et al., 2011), which already constitutes one of the main sources with respect to morbidity and mortality in the developed world. According to the WHO statistics, there are about 1.28 billion adults aged 30–79 years worldwide that suffer from hypertension as a result (Ananda Selva das et al., 2022), much attention has been focused on understanding the pathogenesis and treatment of hypertensive disorders. Some evidence suggests a potential link between gut flora dysbiosis and blood pressure (BP; Li et al., 2017).

In several studies on animal have been illustrated the differences in the gut macrobiotic in animal models of hypertension (Mell et al., 2015), and it compared with the gut macrobiotic in controls (Marques et al., 2017). These studies have used various animal models, including rats with Dahlia sensitivity, rats with spontaneous hypertension, rats with oppressiveness-ii indexed hypertension, and mice treated with Deoxycorticosterone Acetate (DOCA) Salt (Verhaar et al., 2020). The differences in gut microbiota in these models were characterized by a threefold and twofold decrease in bacteria producing propionate and butyrate, respectively, and an increase in the abundance of lactate-producing bacteria (Yang et al., 2015). It will promote an increase in blood pressure. In addition, the abundance of proteobacteria and cyanobacteria increased by approximately 20% (Marques et al., 2017; Huart et al., 2019). Furthermore, the integrity of the intestinal barrier was also found to be affected in animal models of hypertension, as evidenced by increased leakiness, fibrosis, and increased inflammatory markers (Honour, 1982; Yang et al., 2015).

Likewise, similar changes were noted in the gut macrobiota of patients with hypertension. By using 16S ribosomal RNA sequencing, the abundance of lactic acid-producing bacteria was increased (Yang et al., 2015). In addition, it was found that *Firmicutes* accounted for 94.78% of the microbial composition in spontaneously hypertensive rats (SHR), whereas they accounted for only 78.56% in Wistar Kyoto rats (WKY), whereas the abundance of *Bacteroidetes* was approximately 14% higher than in SHR rats, resulting in an increase in the ratio of *Firmicutes* to *Bacteroidetes* (F/B; Yang et al., 2015). And increased F/B ratios are considered a marker of gut ecological dysbiosis (Mariat et al., 2009). Whereas the healthy control’s gut macrobiotic consisted mainly of a gut phenotype enriched with bacteria from the genus Antennae, which is in agreement with the results of the DOCA salt model of hypertension in mice (Santisteban et al., 2017).

Current research increasingly supports the idea that gut microbes regulate blood pressure. Fecal microbiota transplantation experiments have shown that when fecal samples from hypertensive patients and donors with dysbiosis were transferred to non-sensitive mice and rats, both of which had elevated blood pressure (Durgan, 2017). This suggests a reciprocal relationship between gut microbiota and hypertension, highlighting the importance of further investigation of gut microbes to better understand this association (Toral et al., 2019). Therefore, studying gut microbiota could provide valuable insights into the pathogenesis of hypertension and could help to develop related therapeutic strategies.

### 3.2. Gut macrobiotic and atherosclerosis

Like hypertension, atherosclerosis is a major manifestation of cardiovascular disease today. Atherosclerosis (AS) is recognized as a major contributor to coronary artery disease, chronic kidney disease, and peripheral artery disease. AS involves the proliferation and migration of smooth muscle cells, monocytes, and T-lymphocytes, and cholesterol accumulation, among other factors (Gui et al., 2012). Recent research has strongly implicated that the gut microbiota has been strongly implicated with the progression of atherosclerosis (Kasahara et al., 2017; Papandreou et al., 2020). One study found that in patients with coronary artery disease (CAD), the quantity of Lactobacillus puristic was increased while the amount of *Bacteroidetes* was significantly decreased compared to healthy controls. Besides *Bacteroidetes* and *Firmicutes* constitute over 49% of the healthy gut bacterial population (Qin et al., 2010). This suggests that the gut macrobiotic and its metabolites are engaged in the progression of atherosclerosis, and also that dysbiosis of gut flora is linked with systemic inflammation (Drosos et al., 2015).

Patients with atherosclerosis have higher levels of pro-inflammatory gut microbiota (Thursby and Juge, 2017), including *Bacillus*, *Escherichia coli*, *Kielbasi*, *Bifidobacterium* erogenous, *Streptococcus*, and *Lactobacillus salivary* (Arumugam et al., 2011), in contrast to the lower number of macrobiotic involved in the fermentation process, resulted in a reduction in the yield of SCFAs produced from fiber fermentation (Chen et al., 2020). Whereas SCFAs can have anti-inflammatory effects (Aguilar et al., 2014). They can also reduce atherosclerotic plaque formation through modulation and enhanced cholesterol excretion, but the reduced production of SCFAs promotes the progression of atherosclerosis (Bach Knudsen et al., 2018). Future studies should investigate how to intervene in the progression of atherosclerosis by modulating the complex balance of gut microbiota.

### 3.3. Gut macrobiotic and heart failure

Heart failure is a clinical diagnosis caused by dysfunction of the left ventricle, either systolic or diastolic (Takala, 1996). HF is not an independent disease, but rather a late stage in the development of cardiac disease and one of the most major sources of death from cardiovascular disease. Recent researches have reported that the gut microbiota of patients with HF is altered compared to that of healthy individuals (Pasini et al., 2016). Sequencing studies of HF cohorts consistently report lower diversity of the gut microbiota and reduced numbers of specific beneficial microbes in patients with HF (Kamo et al., 2017). According to the “leaky gut” theory, these alterations in gut microbiota may be related to inadequate perfusion to the viscera due to cardiac congestion or impaired ejection capacity in patients with HF, and ischemic edema in the intestine caused by ischemia, which might increase the permeability of the intestinal epithelium (Sandek et al., 2007), thus allowing intestinal macrobiotic and bacterial metabolites to gain access to the circulatory system through the compromised barrier. This can trigger both local and systemic inflammations, It even induces atherosclerosis.

Heart failure is linked to atherosclerosis. There is evidence that circulating levels of pro-inflammatory cytokines such as TNF, IL-1, and IL-6 are increased in patients with heart failure (Tang et al., 2019). Elevated concentrations of these inflammatory factors lead to increased intestinal permeability, higher abundance of pathogenic microorganisms such as *Candida*, *Campylobacter*, and *Shigella*, and a reduction in the abundance of the high-butyrate-producing *Faecalibacterium prausnitzii* (Kamo et al., 2017), which has been shown to contribute to atherosclerotic development by enriching the genes required for the intestinal metabolite, TMAO, in patients with heart failure (Kamo et al., 2017). Moreover, studies have shown that atherosclerosis usually precedes ischemic heart disease (Panizzi et al., 2010), and that atherosclerosis induces myocardial infarction, which leads to a drastic reduction in myocardial contractility, dilation of the left ventricle, and a significant reduction in the heart’s pumping capacity, which can easily lead to heart failure (Swirski and Nahrendorf, 2013). While current research suggests that gut microbiota may have an effect on HF (Conraads et al., 2004), further research is required to determine whether modulating the gut microbiota could relieve heart failure and enhance long-term prognosis (Mann et al., 2004). Later studies should explore potential strategies to treat HF by improving the gut microbiota, such as modulating the complexity and functionality of the intestinal flora through dietary modifications, the employment of probiotics or prebiotics (Hill et al., 2014), and other interventions.

## 4. Metabolic pathway products and cardiovascular disease

### 4.1. SCFAs regulate hypertension

It has already been demonstrated in recent years that regulation of the gut microbiota has an impact on hypertension. Specifically, Yang and others demonstrated a significant imbalance in the microbiota of spontaneously hypertensive rats (SHR) compared to non-sensitive Wistar-Kyoto rats, evidenced by a loss of microbiota species and variety (Yang et al., 2015). This dysbiosis may have a latent effect on the progression of cardiovascular disease through the production of compounds known as short-chain fatty acids. SCFAs interact with host cells through reactions with their receptors.

One manner in which SCFA affects host cells is through interactions with G protein-coupled receptors (GPCRs), particularly Gpr41 and Olfr78. Both receptors interact with SCFAs and are engaged with blood pressure regulation. By vasodilating resistive musculature in an endothelial-dependent manner, Gpr41 has demonstrated anti-hypertensive effects, which could potentially be mediated by Olfr78 (Natarajan et al., 2016). Gpr41 acts as the most potent ligand for propionate and synergizes with propionate to induce vasodilatation, thereby lowering blood pressure (Nutting et al., 1991; Pluznick et al., 2013). Besides mice lacking the Gpr41 gene have been shown to develop hypertension (Zhao et al., 2018). Furthermore, Olfr78 is expressed in renal afferent arterioles, which act as part of the juxtaglomerular apparatus (JGA) and mediate the secretion of renin, which has a key role in the regulation of blood pressure, and the activation of Olfr78 induces the secretion of renin and elevates blood pressure (Pluznick et al., 2013). The impact of propionic acid on epinephrine release was demonstrated through a study by Plunk with others demonstrating the chronic hypertensive effects of SCFA on a group of complex pathways which contribute to the adjustment of BP (Felizardo et al., 2019). In one study, mice maintained on a diet rich in Phyto polysaccharides showed a reproducible, rapid decrease in blood pressure with proportionate use of propionic acid, suggesting acute effects on blood pressure. In contrast, Olfr78−/− mice exhibited a hypertensive response to propionic acid, suggesting that there may be another receptor or receptors mediating this response (Pluznick et al., 2013).

Furthermore, in two models of hypertension, increased levels of lactate-producing bacteria were found in studies on Wisteria Kyoto rats and spontaneously hypertensive rats. Additionally plasma lactate levels were associated with elevated blood pressure in both models. Conversely, the number of bacteria producing butyrate and acetate decreased in both hypertension models. Propionate or acetic treatment protects the host against cardiac injury (Bartolomaeus et al., 2019). And another Gpr41 receptor-mediated SCFA (Kimura et al., 2011), propionate, is shown in wild-type mice to induce vasodilation and an acute hypertensive response.

Regularization of the intestinal macrobiotic may contribute to the emergence of hypertension, also SCFAs may regulate blood pressure through influencing the function of blood pressure-regulating receptors (de la Cuesta-Zuluaga et al., 2018). Therefore, some of the SCFAs might appear to be potentially usable on the therapy of hypertension.

### 4.2. TMA and TMAO affect atherosclerosis

**Figure 3:**
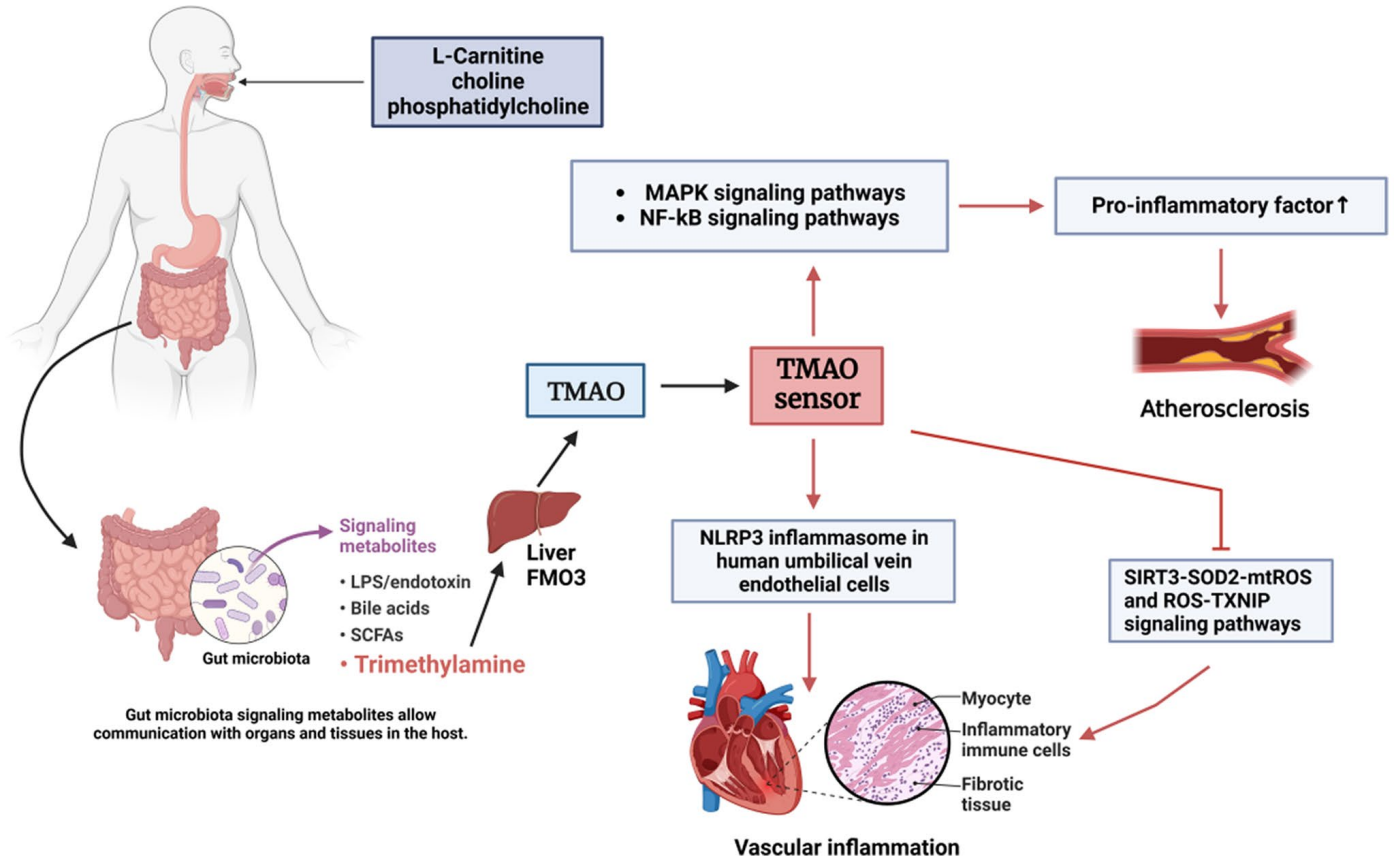
Involvement of TMAO in CVD pathogenesis. TMAO, a metabolite of the gut microbiota, promotes cardiovascular development. The gut microbiota metabolizes complexes containing trimethylamine groups to generate trimethylamine (TMA), which enters the liver *via* the portal circulation and is oxidized to TMAO by FMO3 and binds to the TMAO sensor, which promotes the release of inflammatory factors by affecting the signaling pathways, such as MAPK and NF-κB, and leads to inflammatory responses, which further induces endothelial dysfunction, resulting in atherosclerosis.

Gut microbial metabolites also correlate with the pathogenesis of atherosclerosis. Atherosclerosis, the primary cause of the progression of coronary heart disease and peripheral vascular disease (Cho and Caudill, 2017). For the past few years, it has been shown that TMAO, a metabolizing substance of the intestinal flora, has a potential effect in the development of atherosclerosis (Koeth et al., 2014), Dietary phospholipases such as phosphatidylcholine or carnitine are metabolized by the enzyme complex of the intestinal microbiota to produce TMA (Zeisel and Warrier, 2017), and then TMA is further metabolized by the hepatic enzymes of the host through the portal circulation, and converted in the liver to TMAO (Figure 3; Wang et al., 2011). Patients with atherosclerosis have been found to have less microbial fermentation in their intestines than healthy individuals, but higher levels of LPS, which activates eutrophic bacteria and promotes the release of pro-inflammatory factors that exacerbate the inflammatory response (Bach Knudsen et al., 2018). Also there is evidence that the release of LPS into the bloodstream triggers an immune response. This immune response increases the production of TMA, and studies have found that higher levels of LPS are associated with higher levels of TMAO (Bach Knudsen et al., 2018). Thus high levels of TMA in plasma are also a risk factor for atherosclerosis (Mueller et al., 2015). Furthermore, studies have reported that increased levels of TMAO in the circulation are associated with an elevated risk of CAD-related outcomes (Li et al., 2017).

An animal experiment that sequenced the components of the gut macrobiotic in the 13S rRNA gene in the cecum of mice on a normal food diet and on a intervocalic diet found that oral carnitine ingestion causes atherosclerotic CVD through the microbiota metabolite TMAO (Koeth et al., 2013). In another study, oral choline was administered to several omnivores and vegans without a healthy history of previous illnesses after 2 months, TMAO levels were significantly elevated by blood tests (Zhu et al., 2017), revealing a link between dietary red meat intake and the pathogenesis of atherosclerosis (Figure 3). By studying a mouse model of obstructive sleep apnea, it was shown that suppression of TMA produced ameliorates intermittent hypoxia (IH), which leads to atherosclerosis of the pulmonary arteries (Xue et al., 2017). This indirect evidence suggests that targeting microbiota TMA-cleaving enzymes could serve as a potential treatment for atherosclerosis (From the American Association of Neurological Surgeons (AANS), 2018).

Currently, several researchers have identified potential molecular mechanisms of cardiovascular events related to atherosclerosis and thrombosis (Zhu et al., 2016). For instance, treatment with TMAO was found to lower the capacity of human umbilical vein endothelial cells (HUVECs) compared to the control group, suggesting that TMAO can induce vascular endothelial dysfunction through the activation of the NF-κB pathway (Ma et al., 2017). While atherosclerotic CVD is often correlated with endothelial cell dysfunction, suggesting that TMAO can induce endothelial dysfunction to lead to the development of atherosclerosis (Durpès et al., 2015). Further studies will help to reveal the specific mechanisms linking gut macrobiotic to atherosclerosis and offer new targets for atherosclerosis treatment.

## 5. Targeted therapy for gut macrobiotic

### 5.1. Dietary interventions

As an important regulator of gut microbiota composition, dietary habits are widely believed to have an impact on the composition and function of the gut macrobiota, thereby regulating human health by affecting the digestive and absorptive properties of various nutrients (Xu et al., 2020; Jin et al., 2021). Several studies today have shown that dietary interventions can alleviate and treat a variety of chronic diseases (Org et al., 2017). For example, De Filippis and others have evidenced the cardiovascular health benefits of a diet high in fiber and rich in vegetables and fruits, legumes, and unsaturated fatty acids (Kopp, 2019), A fiber-rich diet promotes the production of acetate macrobiotic, which reduces cardiac hypertrophy and the incidence of CVD. Moreover, other researches have proposed that a Mediterranean diet results in lower TMAO levels and promotes the growth of certain bacteria belonging to the *Firmicutes* and the *Bacteroidetes* (Papadaki et al., 2017), inducing high production of SCFA. On the other hand, diets enriched in phosphatidylcholine and carnitine are tied to elevated levels of TMAO, and lower levels of TMAO are beneficial for the prevention of CVD (Rizzo et al., 2011).

It is known that different gut macrobiotic compositions are linked with different types of cardiovascular disease; therefore, modulating the gut macrobiotic through dietary interventions as a promising therapeutic target in preventing and treating cardiovascular disease. In addition to being less costly than other pharmacological treatments (Grosso et al., 2014). Healthy dietary habits are known to protect against cardiovascular pathogenic factors involving lipid metabolism and inflammation, contributing to the preventive and therapeutic treatment of cardiovascular diseases (De Angelis et al., 2019). In conclusion, influencing the complexity and activity of the gut microbiota by changing dietary habits is a viable approach to preventing and treating cardiovascular disease. However, not all gut microbes are beneficial, as studies have shown that the gut microbial metabolite Levodopa (L-dopa) is capable of decreasing drug availability and causing side effects (Spanogiannopoulos et al., 2016), and accurately identifying “beneficial” and “harmful” microbes remains an important area of interest in the treatment of disease with gut microbes (Maini Rekdal et al., 2019).

### 5.2. Probiotics and prebiotics

Probiotics and prebiotics are also beneficial in the mitigation and treatment of cardiovascular disease. Probiotics are live strains of microorganisms which have been rigorously screened to provide health benefits to their hosts when consumed in the right amounts (Guarner and Schaafsma, 1998; Hill et al., 2014). In addition, prebiotics include oligonucleotide, oligofructose and so on, which act as fermentation substrates stabilizing a population of favorable bacterial flora (Nicolucci et al., 2017). Probiotics and prebiotics, used separately or in combination, have an effect on changes in gut microbiota composition (Gibson et al., 2004). They have been shown to enhance the activity of the beneficial intestinal flora, improve intestinal permeability and blood glucose levels, and alleviate glucose intolerance in patients with diabetes mellifluous.

Probiotics, on the other hand, are a general term for a type of microorganisms beneficial to human intestinal function with specific strains of beneficial bacteria (Gibson et al., 2017), which can establish the balance of the intestinal microbiome, inhibit inflammatory responses, compete for nutrients and specific adherence sites, interfere with pathogenic bacteria (Markowiak and Slizewska, 2017), influence immune regulation, inhibit colonization of pathogenic bacteria (Oelschlaeger, 2010), and maintain normal physiological functions of the intestinal tract, among other benefits.

Specifically, the genera of *Bifidobacterium* and *Lactobacillus* have been widely used, and *Bifidobacterium* has been considered an important probiotic that has an essential effect on maintaining intestinal homeostasis and improving host intestinal health. A study found that cross-feeding between Fecal *coliform* and *Bifidobacterium* adolescence enhanced butyrate formation (Rios-Covian et al., 2015) and reduced risk factors for cardiovascular disease. Gan and others showed that consuming *Lactobacillus rhamnosus* and *Lactobacillus rhamnosus* GR-1 helped minimize left ventricular hypertrophy, enhance left ventricular systolic and diastolic function after myocardial infarction (Chan et al., 2016). Several studies demonstrated that lactobacillus-treated mice exhibited tolerance to hyper-salt-induced hypertension by inhibiting TH17 cells (Wilck et al., 2017), Besides the consumption of *Lactobacillus rhamnosus* was connected with a decrease in cardiovascular risk factors. In addition, a meta-analysis of clinical trials discovered that pro-biotic treatment could powerfully lower total and low-density lipoprotein (LDL) cholesterol levels (Korcz et al., 2018) and improve blood pressure, as well as regulate inflammatory cytokines. Therefore, probiotics have the potential to benefit intestinal health and related disorders, serving as a potential treatment for CVD prevention and lowering the chances of myocardial infarction. Probiotics could potentially be used to prevent coronary artery disease and lower the incidence of CVD (Gan et al., 2014).

### 5.3. Fecal macrobiotic transplantation

Fecal macrobiotic transplantation (FMT) is a curative strategy that involves transplanting beneficial bacteria from a healthy individual’s body to a patient’s digestive tract, restoring a healthy gut microbiota, and suppressing intestinal pathogens (Gallo et al., 2016). On the basis of origin from which transplant-able fecal microbiota is derived, FMT could be classified as either genealogical FMT (using fecal material from a healthy donor) or as autologous FMT (using the patient’s own fecal material). To prepare fecal microbiota for transplantation, different methods such as crude filtering, filtering plus centrifugal (FPC), micro-filtering plus centrifugal (MPC) and clarification can be used. By doing so, FMT can act therapeutically by modulating the host’s gut microbiota (Khanna et al., 2017). One human studies conducted by Vrieze and others demonstrated that FMT transiently increased peripheral insulin sensitivity in patients with metabolic syndrome, which might be linked to elevated rates in the donor’s gut of pre-production bacteria (Vrieze et al., 2014). However, FMT may alter the levels of both harmful and beneficial bacteria in the intestine, potentially leading to adverse effects (Khanna and Pardi, 2016). Additionally, the long-term safety and effectiveness of FMT still require further studies to determine (Dailey et al., 2019). Overall, holds promise as a therapeutic strategy for improving a variety of diseases by modulating the intestinal microbiota. Nevertheless, further research is still necessary to address safety and efficacy concerns and to determine its role in clinical practice.

### 5.4. TMAO reduction therapy

Recent studies have identified many clinical links among the TMAO pathway and adverse CVD outcomes (Jin et al., 2021). Additionally, the deleterious influence of TMAO on the metabolic phenotype of the host brain suggests the need for strategies to suppress TMAO production and eliminate TMAO or its precursors (TMAs; Xu et al., 2020). One strategy that has gained widespread attention is the use of a structural analog of choline called 1,3 dimethylbutanol (DMB), which inhibits the microbiota’s production of TMA from various nutrients, including choline, glycerophosphorylcholine, phosphatidylcholine, and carnitine-related nutrients. By limiting plasma levels of TMAO in high-choline or carnitine diets, DMB has the potential to inhibit the progression of atherosclerosis (Wang et al., 2015). Furthermore, Fujii and others demonstrated that an oral charcoal adsorbent known as AST-120 could effectively eliminate uremic toxins and protect against the development of myocardial hypertrophy and fibrosis progression in models of chronic kidney disease and heart failure (Fujii et al., 2016). These studies suggest that the progression of cardiovascular disease can be limited by suppressing TMAO production and removing TMAO or its precursors, providing evidence for the evolution of future interventions targeting cardiac metabolic phenotype (Astudillo and Mayrovitz, 2021). Nevertheless, more research is required to determine the security and efficacy of such strategies in the clinical setting.

## 6. Conclusion

The gut microbiota forms a commensal association with the human body that is closely linked to human health and disease development. Recent studies have pointed a potential links among the gut microbiota and CVD (Witkowski et al., 2020). First, the gut microbiota could regulate metabolic processes in the human body through gut microbial metabolites as short-chain fatty acids, trimethylamine N-oxide, and bile acids, all of which have been associated with many CAD-related phenotype and can influence the course of cardiovascular disease (Wang and Zhao, 2018). SCFAs can reduce blood cholesterol and triacylglycerol levels and modulate the immune system, thereby reducing the occurrence of atherosclerosis and inflammatory responses (Ott et al., 2006). TMAO promotes the release of inflammatory factors by activating the NF-κB signaling pathway (Seldin et al., 2016), leading to endothelial dysfunction. In addition, the gut microbiota influences thrombosis, as well as vasoconstriction and diastole (Luedde et al., 2017), thus reducing the risk factors for CVD. Cardiovascular disease is strongly influenced by dietary risk factors, and studies have shown that eggs are a major source of dietary cholesterol and that consumption of up to one egg per day can reduce the incidence of CVD (Godos et al., 2021). And with studies finding that Mediterranean Diet (MedDiet) may have anti-inflammatory effects (Casas et al., 2018). MedDiet mainly includes long-chain omega-3 fatty acids from fish and nuts, polyphenols from wine and fruit, probiotics from yogurt products (Chiavaroli et al., 2018). By adding omega-3 s to the diet of hypercholesterolemic rats for 56 days, it was found that the acetylcholine-induced response was mitigated and vascular function was improved in the rats (Mendes Furtado et al., 2022). Research data illustrate that the MedDiet reduces the incidence of CVD by 28% (Chiavaroli et al., 2018). Therefore intervention strategies can be employed to adjust gut microbiota composition and function in CVD patients by dietary modification (David et al., 2014; Verhaar et al., 2020). Studies have shown that increased dietary fiber intake (Cronin et al., 2021), supplementation with prebiotics, probiotics (An et al., 2011; Yoo et al., 2013), and fecal microbiota transplantation (Brandt et al., 2012; Fuentes et al., 2014), which has the potential to improve the therapeutic effects of CVD (Smits et al., 2018), and can influence cardiac metabolism as well as improve inflammatory responses in order to reduce clinical symptoms and disease progression in CVD patients. In addition, researches are underway to develop new drugs targeting the gut microbiota and metabolites for the treatment of cardiovascular disease (Zhou et al., 2020). The gut flora may be able to serve as a new therapeutic target for cardiovascular disease in the future. But further research is required to fully understand the links between the gut microbiota and cardiovascular disease, as well as the underlying mechanisms, offering fresh ideas and approaches for the treatment of cardiovascular disease.

## Author contributions

JZ: Writing – review & editing, Writing – original draft. JL: Writing – review & editing. RZ: Writing – review & editing. GL: Writing – review & editing. SW: Writing – review & editing.

## Funding

The author(s) declare financial support was received for the research, authorship, and/or publication of this article. The research was supported by the grants from: Zhejiang Provincial Program for Medicine and Health (2022KY446, 2022KY447, 2023KY411, 2023KY1345, 2023KY1347), Key Laboratory of Precision Medicine for Atherosclerotic Diseases of Zhejiang Province, China (2022E10026), Social Development Science and Technology Foundation of Taizhou (21ywb115, 21ywb118, 20ywb143), Social Development Science and Technology Foundation of Wenling (2020S0180083, 2021S00156, 2021S00197, 2020S0180127).

## Conflict of interest

The authors declare that the research was conducted in the absence of any commercial or financial relationships that could be construed as a potential conflict of interest.

## Publisher’s note

All claims expressed in this article are solely those of the authors and do not necessarily represent those of their affiliated organizations, or those of the publisher, the editors and the reviewers. Any product that may be evaluated in this article, or claim that may be made by its manufacturer, is not guaranteed or endorsed by the publisher.
